# Heterotrophic Cultivation of the Cyanobacterium *Pseudanabaena* sp. on Forest Biomass Hydrolysates toward Sustainable Biodiesel Production

**DOI:** 10.3390/microorganisms10091756

**Published:** 2022-08-30

**Authors:** Dimitra Karageorgou, Alok Patel, Ulrika Rova, Paul Christakopoulos, Petros Katapodis, Leonidas Matsakas

**Affiliations:** 1 Laboratory of Biotechnology, Department of Biological Applications and Technologies, University of Ioannina, 45100 Ioannina, Greece; 2 Biochemical Process Engineering, Division of Chemical Engineering, Department of Civil, Environmental, and Natural Resources Engineering, Luleå University of Technology, SE-971 87 Luleå, Sweden

**Keywords:** cyanobacteria, *Pseudanabaena* sp., wood biomass, heterotrophic growth, lipid production, organosolv pretreatment, biodiesel

## Abstract

Environmental pollution, greenhouse gas emissions, depletion of fossil fuels, and a growing population have sparked a search for new and renewable energy sources such as biodiesel. The use of waste or residues as substrates for microbial growth can favor the implementation of a biorefinery concept with reduced environmental footprint. Cyanobacteria constitute microorganisms with enhanced ability to use industrial effluents, wastewaters, forest residues for growth, and concomitant production of added-value compounds. In this study, a recently isolated cyanobacterium strain of *Pseudanabaena* sp. was cultivated on hydrolysates from pretreated forest biomass (silver birch and Norway spruce), and the production of biodiesel-grade lipids was assessed. Optimizing carbon source concentration and the (C/N) carbon-to-nitrogen ratio resulted in 66.45% *w*/*w* lipid content when microalgae were grown on glucose, compared to 62.95% and 63.79% *w*/*w* when grown on spruce and birch hydrolysate, respectively. Importantly, the lipid profile was suitable for the production of high-quality biodiesel. The present study demonstrates how this new cyanobacterial strain could be used as a biofactory, converting residual resources into green biofuel.

## 1. Introduction

The projected increase in demand for fossil fuels (37% by 2040), together with their rising price, dwindling reserves, and environmental costs, has triggered the search for alternative, sustainable, and renewable fuel sources [1,2], such as ethanol, butanol, hydrogen, methanol, and bio-oil [3]. This is particularly true in the EU, which aims to replace traditional fuels with biofuels by 2045 [4]. Biodiesel consists of one of the available solutions that can be used to offset CO_2_ emissions [5]. First-generation biodiesel—produced mainly from food crops—has encountered strong criticism due to the use of fertile land for fuel rather than food production. Second-generation biodiesel has overcome this disadvantage as nonedible biomass is used, but diesel production is not cost-effective [3,6].

Microorganisms, especially microalgae and cyanobacteria, represent promising candidates for sustainable and cheaper second-generation biofuels. Cyanobacteria are often referred to as blue-green microalgae [7]. They can grow at high rates, without the need for arable land or potable water, not competing for the production of food. Both microalgae and cyanobacteria are able to produce a plethora of different metabolites, including proteins, carbohydrates, exopolysaccharides, and lipids [1,8]. Some microalgae are considered oleaginous microorganisms, as a greater rate than 20% *w*/*w* of their cell biomass can be constituted by lipids [9]. In a biorefinery concept, the simultaneous production of more than one metabolite is desirable [10]. For biodiesel production, biomass and lipid productivity and the lipid profile play important roles in defining the efficacy of the system. Their rapid growth rate, high amount of biomass and lipids, and ability to bind and use CO_2_ make them strong candidates for biodiesel production [11]. Both cyanobacteria and microalgae adapt well to different environments, exploiting the available nutrient sources and producing useful metabolites [7]. To this end, filamentous cyanobacteria are capable of removing nutrients, thus reducing the organic load of waste streams and contributing to an environmentally friendly recycling process [12].

Most of the oleaginous microalgae and cyanobacteria produce lipids containing 4–28 carbons, which include saturated, monounsaturated, or polyunsaturated fatty acids. Strains that produce large amounts of saturated and monounsaturated fatty acids are better candidates for biodiesel production. In contrast, the susceptibility of polyunsaturated fatty acids to autoxidation makes them unsuitable for this purpose [13].

Cyanobacteria can grow under autotrophic, mixotrophic, or heterotrophic conditions [14]. While glucose is the most widely used substrate, sustainable and renewable substrates would lower production costs [15]. Suitable low-cost substrates include crude grease, cassava, and sweet sorghum [16,17,18]. Another untapped substrate is lignocellulosic biomass, which is obtained from agricultural and forestry residues/waste. A pretreatment step is necessary to make the carbohydrate fractions (i.e., cellulose and secondary hemicellulose) more accessible to enzymes and in turn saccharify the insoluble polysaccharides to monomeric sugars [19,20,21]. The use of waste or residual streams as a carbon and/or nitrogen source in biorefinery processes can not only reduce the amounts of those residues, but also generate renewable green products such as biodiesel.

The forestry industry accounts for a large share of the Swedish economy, as over 50% of the country is covered with forests [21] rich in Norway spruce (*Picea abies*), Scots pine (*Pinus sylvestris*), and birch (*Betula pendula*, *Betula pubescens*). Therefore, the residues of these trees could be used as a carbon source for the cultivation of microorganisms, and the resulting lipids might be converted into biodiesel.

In the current study, we used the filamentous cyanobacterium *Pseudanabaena* sp. strain Pamv7 isolated from Lake Pamvotis, in Greece, as a cell factory for the conversion of hydrolysates from residual forest biomass into biodiesel. Lake Pamvotis is located in NW Greece (Ioannina, Greece (39°39′45″ N 20°53′06″ E) and constitutes a natural habitat for a plethora of filamentous cyanobacteria species [22]. Specifically, the study aimed to evaluate the heterotrophic growth of *Pseudanabaena* sp. on sugars, optimize biomass and lipid accumulation, and use sugars from saccharified birch and spruce biomass as substrate. To further estimate the potential of the produced lipids to assist as feedstock for biodiesel production, the quality of the biodiesel was assessed by estimating biodiesel properties.

## 2. Experimental

### 2.1. Strain and Cultivation Conditions

*Pseudanabaena* sp. strain Pamv7 (Taxonomy ID: 2587810 in NCBI website) cells were cultivated autotrophically in BG-11 medium (Fluka Analytical, Sigma-Aldrich, St. Louis, MO, USA) aerobically at 25 °C and subjected to a 12 h/12 h light/dark period with white fluorescent irradiation. The intensity of light during the light period was 80 μmol photons/m^2^·s. For heterotrophic cultivation, *Pseudanabaena* sp. cells were harvested via centrifugation at 5000 rpm for 10 min, washed twice with distilled water, and suspended in 250 mL Erlenmeyer flasks containing 100 mL of BG-11 medium enriched with yeast extract and glucose (20 g/L) as nitrogen and carbon sources, so as to achieve the desired C/N ratio. The cultures were incubated in the dark with continuous shaking (100 rpm) at 25 ± 1 °C, and they were used as inoculum for the main cultures.

The effect of various C/N ratios on both biomass and lipid synthesis was evaluated by growing cells in BG-11 medium with a stable glucose concentration of 20 g/L and varying yeast extract concentrations adjusted to achieve C/N ratios of 20, 40, 60, 80, and 100. Knowing the nitrogen concentration in BG-11 and analyzing the total nitrogen concentration of yeast extract using the Kjeldahl method, i.e., 11% of total weight, we calculated the amount of yeast extract needed for the above ratios. To investigate how biomass and lipid production are impacted by the initial glucose content, cells were grown at C/N 40 and supplemented with 20 g/L, 40 g/L, 60 g/L, 80 g/L, and 100 g/L glucose. Cell growth on renewable glucose sources was tested by diluting the appropriate volume of birch or spruce hydrolysate in BG-11 medium to obtain a glucose concentration of 20 g/L, whereas yeast extract was added to ensure a C/N ratio of 40.

In all cultures, the pH of the medium was adjusted to 6.7, and all flasks and media were autoclaved. The inoculum constituted 10% *v*/*v* of the culture and was taken from heterotrophic cultures in exponential phase, after the determination of biomass concentration and a microscopic check for contamination. The cultures were grown in the dark, with continuous shaking (100 rpm), at 25 ± 1 °C. Every 24 h, samples were taken under sterile conditions to monitor growth, biomass and lipid production, and sugar consumption.

### 2.2. Preparation and Enzymatic Saccharification of Organosolv/Steam Explosion-Pretreated Birch and Spruce Biomass

Wood biomass was subjected to hybrid organosolv/steam explosion pretreatment [23,24], which resulted in the fractionation of woody biomass into pretreated solids with high cellulose content, as well as lignin and hemicellulose fractions. Briefly, birch (*B. pendula*) and spruce (*P. abies*) woodchips were milled to less than 1 mm particles with the help of a Retch SM 300 knife mill (Retsch GmbH, Haan, Germany). The hybrid organosolv/steam explosion was performed at 200 °C using ethanol in water solutions (60% *v*/*v* and 52% *v*/*v* for birch and spruce, respectively) and 1% *w*/*w*_biomass_ H_2_SO_4_ for 15 min and 30 min, respectively [23,24]. The pretreated biomass was separated from the liquid by vacuum filtration, washed with ethanol, and dried at room temperature. The pretreatment liquid was collected for the recovery of lignin and solubilized hemicellulose (not used in the current study). The pretreated biomass was subjected to enzymatic saccharification, whereby a 10% *w*/*w* biomass solution was prepared in 50 mM citrate phosphate buffer at pH 5. Cell CTec2 (Novozymes A/S, Boswell, Denmark) at 20 FPU/g of dry biomass was used for enzymatic saccharification for 24 h at 50 °C and shaking (180 rpm). The resulting hydrolysate was separated from the remaining solids by centrifugation, and the amount of sugars (specifically glucose) was determined by high-performance liquid chromatography (HPLC).

### 2.3. Estimation of Dry Biomass and Biomass Productivity

Cyanobacterial biomass was determined gravimetrically and expressed as dry biomass (g/L). Specifically, the samples were centrifuged at 5000 rpm for 10 min, washed twice with distilled water, resuspended in distilled water, and finally filtered under vacuum through pre-weighted 0.45 μm Whatman membranes. The filtered samples were dried at 60 °C until they reached a consistent weight, and the dried biomass was weighed. Biomass concentration in the cultivation medium was calculated on the basis of the difference in weight of the membrane before and after filtration. The following equation was used to compute biomass productivity (P; g/L/day):

P = (X2 − X1)/(t2 − t1),(1)

where X2 and X1 correspond to the biomass weights (g/L) at the start of cultivation (day t1) and the completion of cultivation (day t2), respectively. By measuring absorbance at 590 nm, growth was assessed.

### 2.4. Determination of Total Lipid Concentration, Lipid Content, and Lipid Productivity

For lipid extraction, we used a modified version of Folch’s method [25]. Specifically, the dried cell biomass was initially powdered with a mortar, to facilitate lipid extraction, followed by addition of a mixture of chloroform/methanol (2:1 *v*/*v*) and incubation overnight under mild shaking. The next day, water was added, and the mixture was centrifuged, creating two phases with the cell pellet in the middle. The lower chloroform phase with the extracted lipids was transferred to pre-weighed glass vials, which were dried. Lipid weight was determined gravimetrically, and the lipid concentration (g/L) was calculated on the basis of the initial sample. Lipid content (Y; % *w*/*w*) was calculated using the following equation:

Y = (LC/BC) × 100,(2)

where LC is the lipid concentration (g/L), and BC is the biomass concentration (g/L). Productivity (P; mg/L·day) was calculated using the following equation:

P = (Z2 − Z1)/(t2 − t1),(3)

where Z2 and Z1 are the lipid weights (g/L) at two different cultivation times, corresponding to days t1 (start point of cultivation) and t2 (endpoint of cultivation).

### 2.5. Determination of Residual Sugars

Sugars were determined using an HPLC system equipped with a refractive detector and an Aminex HPX-87P column (Bio-Rad, Hercules, CA, USA). The column was maintained at 85 °C.

### 2.6. Analysis of the Lipid Profile and Estimation of Biodiesel Properties

The extracted lipids were transesterified with acid catalyst according to Wychen et al. (2013) [26]. Initially, 50–100 mg of lipids were mixed with 2 mL of chloroform/methanol solution (2:1, *v*/*v*) in ace pressure tubes (Sigma Aldrich), followed by the addition of 3 mL of 0.6 M HCl/methanol. The tubes were placed in a preheated water bath at 85 °C for 1 h. Once the transesterification reaction was complete, the mixture was cooled at room temperature, and 3 mL of *n*-hexane was added. The mixture was centrifuged at 8000 rpm (7881× *g*) for 10 min to separate the different layers. The upper *n*-hexane layer containing the fatty acid methyl esters was aspirated and transferred to new gas chromatography vials for analysis on a Clarus 690 gas chromatographer (PerkinElmer, Waltham, MA, USA) coupled to a mass spectrometer (Clarus SQ8; PerkinElmer) and equipped with a capillary column (Elite-FFAP; 30 m, 0.25 mm ID, 0.25 µm df, Cat. # N9316352; PerkinElmer). Analysis conditions were as in our previous study [27]. The fuel properties of the obtained lipids were assessed as described earlier [28,29].

## 3. Results and Discussion

### 3.1. Effect of the C/N Ratio on Biomass and Lipid Production in Pseudanabaena sp.

Cyanobacterial strains are able to produce biomolecules in various quantities such as lipids, proteins, and carbohydrates, adjusting their metabolisms to different medium compositions. Nutrient concentration is a critical factor affecting lipid accumulation in microorganisms such as microalgae and cyanobacteria [22].

Cell growth, lipid concentration and profile are affected by the C/N ratio and the nature of the nitrogen source. In general, cyanobacteria can use various nitrogen sources, including sodium nitrate, yeast extract, urea, and ammonia, with complex sources such as yeast extract, rich in amino acids and vitamins, giving better results [30]. Some studies have proposed the use of a two-stage cultivation system, whereby, in the first stage, the environment is optimal for biomass accumulation (normally low C/N ratio), while, in the second stage, nitrogen starvation favors the production and accumulation of lipids [31,32].

As it is known that the medium’s components and their ratios influence biomass and lipid accumulation in oleaginous microorganisms [33], we initially examined the effect of different C/N ratios (20–100) while fixing the glucose concentration (20 g/L) and varying the concentration of nitrogen.

**Figure 1:**
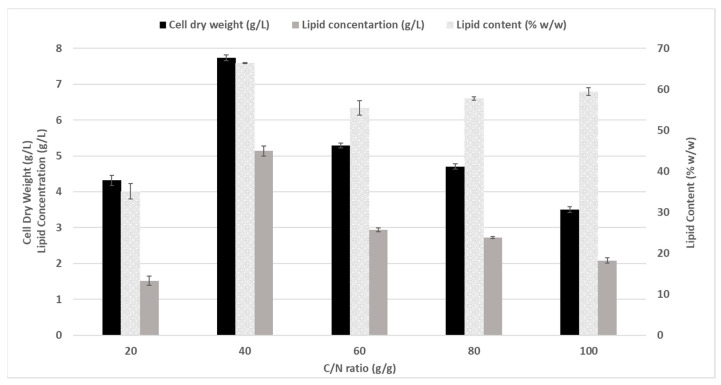
Growth and lipid accumulation of *Pseudanabaena* sp. at different C/N ratios. The effect of C/N ratios on cell dry weight (g/L), lipid content (% *w*/*w*), and total lipid concentration (g/L) is shown after 120 h of culture. All measurements were performed in triplicate; error bars denote the standard deviation.

As presented in Figure 1, when the C/N ratio changed from 20 to 40, an increase in cell dry weight was observed, peaking at 7.74 ± 0.08 g/L, but decreasing gradually thereafter. Similarly, lipid content almost doubled when the C/N ratio increased from 20 (35.15% ± 0.13%) to 40 (66.45% ± 0.14%); for C/N ratios above 40, it varied between 55.45% and 59.45% *w*/*w*. Lastly, the lipid concentration also followed a similar trend, increasing from 1.52 ± 0.13 g/L to 5.14 ± 0.14 g/L when the C/N ratio changed from 20 to 40, but then gradually declined. These results suggest that, at a low C/N ratio, nitrogen exerted an inhibitory effect; however, as the C/N ratio increases from 20 to 40, both biomass and lipid formation experienced an increase. It is reported that nitrogen limitation enhances lipid accumulation in oleaginous microorganisms [34]. Above an optimal C/N ratio of 40, the lipid content exhibited a slight increase as the C/N ratio increased from 60 to 100, while a negative impact on biomass production and lipogenesis was observed. The effect of nitrogen concentration on both biomass and lipid production has been reported many times. For instance, *Phormidium autumnale* cells were able to accumulate from 0.93% to 20.58% *w*/*w* lipid content under different nitrogen sources and C/N ratios [32], while *Hematococcus pluvialis* and *Neochloris oleoabundans* could enhance their lipid contents from 15% to 33% *w*/*w* and from 23% to 37% *w*/*w*, respectively, by changing the nitrogen content [35,36].

Accordingly, C/N 40 and yeast extract as a nitrogen source were selected for further experiments.

### 3.2. Effect of Initial Glucose Concentration on Lipid Production and Accumulation in Pseudanabaena sp.

In the next stage, we examined the effect of the initial glucose concentration on the production of both biomass and lipid of *Pseudanabaena* sp. Glucose constitutes the preferred carbon source for the heterotrophic cultivation of microorganisms, owing to its elevated energy content (2.8 kJ/mol) [34] and ease of assimilation. Here, we tested various concentrations of glucose, ranging from 10 g/L to 100 g/L, in BG-11 medium containing yeast extract to achieve a C/N ratio of 40.

The effect of glucose concentration on cell dry weight, lipid content, lipid concentration, biomass, and lipid yield, as well as the residual glucose concentration, is summarized in Table 1. The highest biomass (7.74 ± 0.08 g/L) was achieved at 20 g/L glucose. Even though the biomass yield was slightly higher at 10 g/L (0.435 g/g_substrate_), the overall biomass and lipid production were higher at 20 g/L. At initial glucose concentrations of 40 g/L and above, biomass formation, biomass formation yield, and lipid accumulation showed a progressive and significant decline. This pattern indicates that higher glucose concentrations exerted an inhibitory effect on the cells, as also suggested by the increasing concentration of unconsumed glucose in the medium. On the basis of these results, 20 g/L was the optimal initial concentration of glucose, corresponding to a lipid concentration of 5.14 ± 0.12 g/L (66.45% ± 0.94% *w*/*w* lipid content) and a lipid formation yield of 0.274 ± 0.005 g/g_substrate_. Taking into account those results, the initial glucose concentration of 20 g/L was used for further experiments. With other cyanobacterial or microalgal species, it has been shown that biomass and bio-compound production depend not only on the initial carbon source concentration, but also on the nature of the carbon source or other medium components, the culture conditions, and the strain of the microorganism. For instance, *Phormidium* sp. could produce 1.18 g/L and 5.54 g/L cell dry biomass when cultivated on sucrose and fructose, respectively [22,37], while *Anabaena variabilis* could produce 10 g/L biomass compared to the 0.41 g/L of *Nostoc flagelliforme*, both using fructose [12,38,39].

### 3.3. Time Course of Pseudanabaena sp. Grown on Birch and Spruce Biomass Hydrolysates

After optimizing the biomass and lipid production and accumulation of the cyanobacterium on commercial glucose, the potential of replacing it with glucose derived from wood residues was investigated. For this purpose, we used birch and spruce hydrolysates from pretreated biomass. The pretreated solids were enzymatically saccharified, yielding 81.75 g/L of glucose for birch hydrolysate (BH) and 58.51 g/L for spruce hydrolysate (SH), which corresponded to 94.5% and 73.1% cellulose hydrolysis, respectively. The hydrolysates were then diluted with BG-11 medium to an initial glucose concentration of 20 g/L.

**Figure 2:**
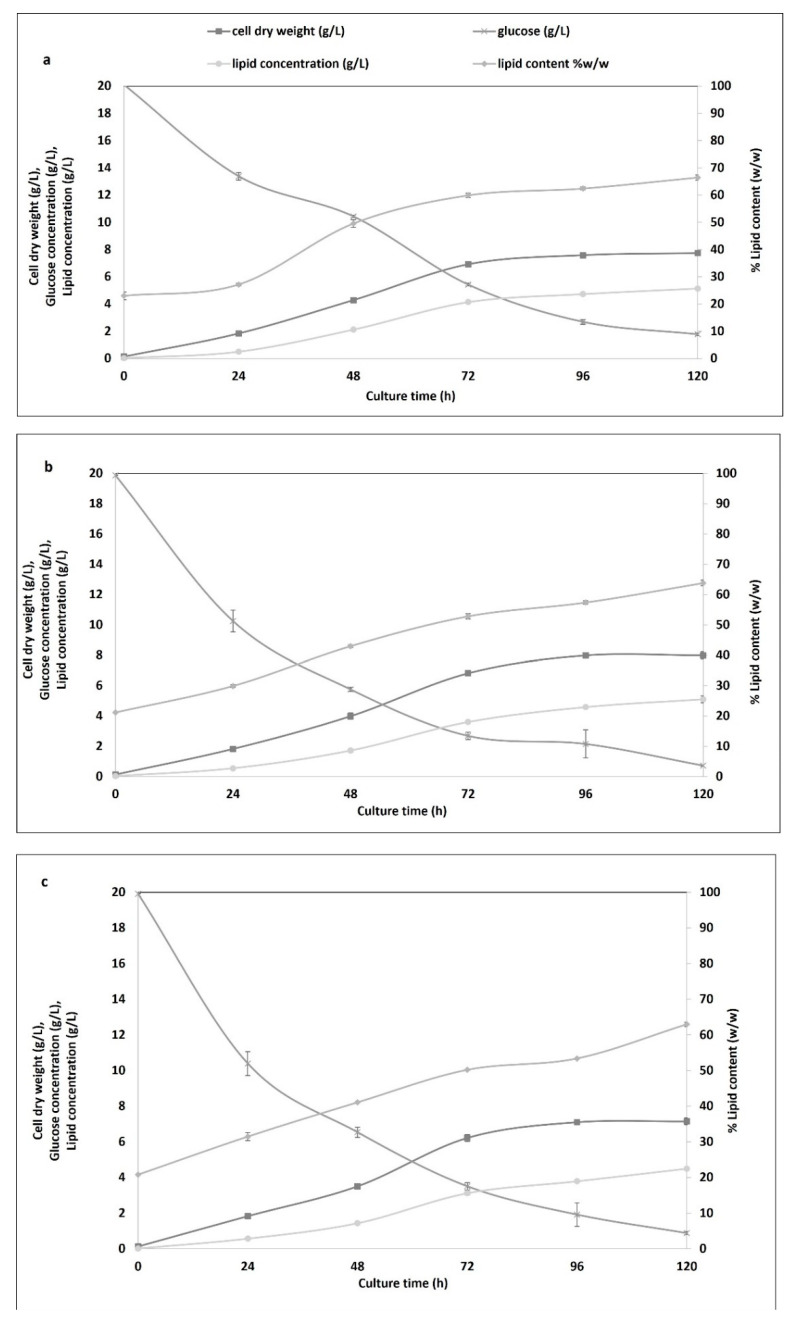
Time course showing batch cultivation of *Pseudanabaena* sp. in (**a**) glucose, (**b**) birch hydrolysate, and (**c**) spruce hydrolysate with 20 g/L glucose and C/N 40. All measurements were performed in duplicate and are represented as average values with standard deviation.

Time course changes in cell dry weight, lipid content, lipid concentration, and glucose consumption are presented in Figure 2, whereas Table 2 shows the biomass, lipid productivity, and the corresponding yields in comparison to those growing on glucose. Both cell dry weight and lipid content were almost identical in all cases at the expiry of the cultivation time (120 h). Specifically, cell dry weight was 7.74 ± 0.08 g/L, 7.98 ± 0.25 g/L, and 7.15 ± 0.20 g/L, while lipid content was 66.45% ± 0.94%, 63.79% ± 0.95%, and 62.95% ± 0.64% *w*/*w* in the medium containing glucose, BH, and SH, respectively. The biomass and lipid productivity that were obtained when cells were cultivated in BH and SH were similar to when glucose was the carbon source, which demonstrates the suitability of these renewable sources for cyanobacterial growth. The residual glucose concentration at the end of the cultivation period was 1.77 ± 0.09 g/L, 0.72 ± 0.04 g/L, and 0.88 ± 0.08 g/L, respectively, indicating near-complete consumption.

In all cases, the exponential growth phase lasted from 24 to 90 h (Figure 2), and it coincided with the period of highest biomass and lipid production, as well as glucose consumption. Similar cell growth, lipid production, and glucose consumption dynamics were observed irrespective of whether the substrate was pure glucose, BH, or SH. There are previous studies indicating the instant insertion of cells into the exponential phase from the early hours of culture [12].

This finding indicates that the hydrolysates were devoid of inhibitors, making them a suitable feedstock for microbial cultivations. From all the above results, it is evident that *Pseudanabaena* sp. is capable of metabolizing glucose toward lipids irrespective of the glucose origin. It has been proposed that oleaginous microorganisms are usually capable of using the carbon source from their cultural medium, irrespective of its origin [15]. The use of forest biomass from *Pseudanabaena* sp. cells for lipid production that can serve as feedstock for biodiesel is of high importance.

### 3.4. Fatty Acid Profiles of Pseudanabaena sp. Grown on Glucose or Birch/Spruce Hydrolysates

The majority of lipids synthesized by oleaginous microorganisms are in the form of triacylglycerides (TAGs), and their length ranges from C_14_ to C_24_. These TAGs can be transesterified into a mixture of fatty esters that constitutes biodiesel. To examine whether the lipids obtained from *Pseudanabaena* sp. grown on glucose, BH, and SH had a fatty acid profile suitable for biodiesel production, their fatty acid composition was analyzed. As shown in Table 3, six different fatty acids, including saturated and monounsaturated fatty acids, were produced. Saturated fatty acids accounted for about 73%, 74%, and 77% of all fatty acids in cells grown on BH, SH, and glucose, respectively. Among them, C_16:0_ was the most abundant fatty acid, with a content of about 40%, followed by C_18:0_ with about 30%. Notably, cells grown on hydrolysates also synthesized C_14:0_, which was absent from glucose-grown cells. In comparison, C_14:1_, which is derived from C_14:0_ via addition of a double bond by a desaturase, presented the same content in all cases. Similarly, C_16:0_ accounted for 44% of saturated fatty acids on glucose and 40% on hydrolysates, whereas the C_16:1_ content was identical in all samples. C_18:0_ content was 32.82% in glucose-grown cells, but dropped to 29.02% and 30.05% in SH- and BH-grown cells, respectively. This decrease was offset by an increase in C_18:1_, which augmented from 11.5% in glucose-grown cells to 14.19% and 15.77% in SH- and BH-grown cells, respectively.

Cyanobacteria can produce significant amounts of lipids, usually with a chain length ranging from C_14_ to C_18_ [40]. The high amount of saturated and monounsaturated fatty acids with the absence of long aliphatic fatty acids in cyanobacterial strains such as in *Limnothrix* sp., makes them appropriate candidates for biodiesel production [8]. C_16:0_ is the most abundant fatty acid in several cyanobacterial species, whereas C_14:0_, C_14:1_, C_16:1_, and C_18:1_ are present in much lower amounts [41]. The same results were obtained in the present study, whereby C_16:0_ was the dominant fatty acid, followed by C_18:0_ and then C_18:1_, C_14:1_, C_16:1_, and C_14:0_. C_16:0_ is synthesized on the cytosolic face of the endoplasmic reticulum, where longer fatty acids are then produced through elongation and desaturation steps. C_18:1_ is derived from C_18:0_ by the addition of a double bond in a late desaturation step that requires NADPH, NADH, and oxygen [42], which is the last common intermediate before synthesis splits between the omega-3 and omega-6 pathways.

The aim of this work was to generate bioenergy in the form of biodiesel through the use of microbially produced lipids. As revealed by the fatty acid profile, the resulting lipids were composed mainly of saturated and monounsaturated fatty acids, which favors biodiesel production. Polyunsaturated fatty acids, which lower biodiesel stability [43], were absent from the presently produced lipids.

### 3.5. Estimation of Biodiesel Properties

Biodiesel properties depend on the chemical composition of the feedstock used [44]. Fatty acid chain length and the presence of unsaturation are important factors in determining the physicochemical characteristics of biodiesel [45]. Biodiesel must meet international standards, such as ASTM D6751 (USA) and EN 14214 (Europe). A high cetane number, which leads to lower NOx emissions, can be achieved by using feedstocks rich in long-chain saturated fatty acids [46]. The CN of the lipids obtained from glucose and hydrolysates were similar, and all of them were higher than the minimum requirements (Table 4).

The saturation of fatty acids is a key element in determining the kinematic viscosity of biodiesel, which increases with increasing chain length and saturation [47]. Double bonds present in fatty acid chains react with oxygen when exposed to air. Viscosity increases at low temperature, but also with increasing chain length or saturation of fatty acids, meaning that the degree of unsaturation, as well as the location and number of double bonds, severely affects the fluidity of biodiesel. The viscosity of unsaturated fatty acids is also dependent on the number and nature of double bonds (*cis* or *trans*) but is less affected by position [40]. The kinematic viscosity estimated for the lipids obtained in the current work falls within the limits set by the international standards (Table 4). Lastly, density plays a crucial role in determining the fuel injection properties of biodiesel. Density is limited to 0.860–0.900 g/cm^3^ at 15 °C by EN 14214 standards, while no specification for density exists in the ASTM D6751 standard. All biodiesels obtained in this study displayed an optimum density in line with EN 14214 guidelines.

The abundance of saturated fatty acids in fatty acid methyl esters reduces the autoxidation of biodiesel, extending its shelf life, whereas a high amount of unsaturated fatty acids increases its cold flow plugging properties. Hence, to control fuel properties, the ratio of saturated to unsaturated fatty acids needs to be optimized [23]. Here, this ratio was roughly 74:26 (Table 4), which can be considered optimal for biodiesel.

## 4. Conclusions

In this study, we investigated the potential of using forest hydrolysates as a carbon source for lipid production by the newly isolated cyanobacterium *Pseudanabaena* sp. (Pamv7). Use of hydrolysates from pretreated forest biomass as a feedstock for cyanobacterial cultivation has numerous environmental benefits as it can reduce the ecological footprint of the process and support large-scale production of biodiesel. In this study, forest hydrolysates were generated via enzymatic hydrolysis of organosolv/steam explosion-pretreated birch and spruce. When the cyanobacterium was cultivated heterotrophically on the hydrolysates with 20 g/L glucose and C/N 40, lipid production reached 5.09 g/L and 4.50 g/L for birch and spruce hydrolysates, respectively. These lipids consisted of a mixture of saturated and monounsaturated fatty acids, and they were shown to meet international standards for biodiesel quality.

## Figures and Tables

**Table 1:** Growth of *Pseudanabaena* sp. at various initial glucose concentrations.

Initial Glucose Concentration (g/L)	Cell Dry Weight (g/L)	Lipid Content (% *w*/*w*)	Lipid Concentration (g/L)	Biomass Yield (g/g _substrate_)	Lipid Yield (g/g_substrate_)	Residual Glucose Concentration (g/L)
10	4.30 ± 0.08	40.50 ± 0.99	1.74 ± 0.08	0.435 ± 0.008	0.176 ± 0.007	0.11 ± 0.02
20	7.74 ± 0.08	66.45 ± 0.94	5.14 ± 0.12	0.425 ± 0.005	0.274 ± 0.005	1.77 ± 0.09
40	5.68 ± 0.18	43.05 ± 0.78	2.44 ± 0.03	0.242 ± 0.018	0.104 ± 0.006	16.49 ± 1.03
60	5.53 ± 0.10	45.70 ± 0.14	2.53 ± 0.02	0.193 ± 0.010	0.088 ± 0.005	31.37 ± 1.94
80	4.58 ± 0.04	41.10 ± 1.84	1.88 ± 0.09	0.125 ± 0.005	0.051 ± 0.004	43.32 ± 1.04
100	3.62 ± 0.11	37.00 ± 2.40	1.34 ± 0.13	0.109 ± 0.010	0.041 ± 0.006	66.75 ± 2.01

**Table 2:** Biomass and lipid synthesis in heterotrophically cultivated *Pseudanabaena* sp.

	Glucose	Birch Hydrolysate	Spruce Hydrolysate
**Cell Dry Weight (g/L)**	7.74 ± 0.08	7.98 ± 0.25	7.15 ± 0.20
**Lipid Content (% *w*/*w*)**	66.45 ± 0.94	63.79 ± 0.95	62.95 ± 0.64
**Lipid Concentration (g/L)**	5.14 ± 0.12	5.09 ± 0.23	4.50 ± 0.08
**Biomass Productivity (g/L** **·** **day)**	1.52 ± 0.07	1.57 ± 0.05	1.43 ± 0.06
**Lipid Productivity (mg/L** **·** **day)**	1028 ± 25	1013 ± 26	900 ± 17
**Residual Glucose Concentration (g/L)**	1.77 ± 0.09	0.72 ± 0.04	0.88 ± 0.08
**Biomass Yield (g/g_substrate_)**	0.425 ± 0.005	0.414 ± 0.014	0.374 ± 0.005
**Lipid Yield (g/g_substrate)_**	0.282 ± 0.005	0.264 ± 0.013	0.235 ± 0.002

**Table 3:** Fatty acid profile of lipids obtained from heterotrophically cultivated *Pseudanabaena* sp. and analyzed by gas chromatography.

Fatty Acids	Glucose		Birch Hydrolysate		Spruce Hydrolysate	
**Saturated Fatty Acids**						
**C14:0**	-		3.92%		3.70%	
**C16:0**	43.87%	76.69%	39.78%	72.71%	40.35%	74.10%
**C18:0**	32.82%		29.02%		30.05%	
**Monounsaturated Fatty Acids**						
**C14:1**	7.54%		7.51%		7.33%	
**C16:1**	4.24%	23.31%	4.01%	27.29%	4.38%	25.90%
**C18:1**	11.53%		15.77%		14.19%	

**Table 4:** Estimation of physicochemical properties of biodiesel obtained by cyanobacterial growth on glucose or hydrolysates.

Biodiesel Properties	Units	Glucose; C/N 40	Birch Hydrolysate; C/N 40	Spruce Hydrolysate; C/N 40	Biodiesel Standards	
					ASTM D6751	EN 14214
**Saturated Fatty Acids**		76.69	72.716	74.1		
**Monounsaturated Fatty Acids**		23.31	27.29	25.9		
**DU**		23.31	27.29	25.9		
**Long-Chain saturation factor**	-	20.797	18.488	19.06	-	-
**Density**	g/cm^3^	0.861	0.863	0.862	-	0.86–0.90
**Cold filter plugging point**	°C	48.863	41.609	43.401	-	-
**Cloud point**	°C	18.084	15.932	16.232		
**Pour point**	°C	12.81	10.474	10.8		
**Cetane number**	-	68.991	68.067	68.286	47 min	51 min
**Kinematic viscosity**	mm^2^/s	4.055	3.982	3.992	1.9–6.0	3.5–5.0
**Saponification value**	mg KOH/g-oil	210.115	211.068	211.213	0.50 min	0.50 min
**Iodine value**	mg I_2_/100 g	14.601	18.185	17.133	-	120 max
**High Heating value**	MJ/kg	39.241	39.191	39.213	-	-

max, maximum; min, minimum; -, not reported.
